# Waiting Endurance Time Estimation of Electric Two-Wheelers at Signalized Intersections

**DOI:** 10.1155/2014/702197

**Published:** 2014-05-07

**Authors:** Mei Huan, Xiao-bao Yang

**Affiliations:** MOE Key Laboratory for Urban Transportation Complex Systems Theory and Technology, Beijing Jiaotong University, Beijing 100044, China

## Abstract

The paper proposed a model for estimating waiting endurance times of electric two-wheelers at signalized intersections using survival analysis method. Waiting duration times were collected by video cameras and they were assigned as censored and uncensored data to distinguish between normal crossing and red-light running behavior. A Cox proportional hazard model was introduced, and variables revealing personal characteristics and traffic conditions were defined as covariates to describe the effects of internal and external factors. Empirical results show that riders do not want to wait too long to cross intersections. As signal waiting time increases, electric two-wheelers get impatient and violate the traffic signal. There are 12.8% of electric two-wheelers with negligible wait time. 25.0% of electric two-wheelers are generally nonrisk takers who can obey the traffic rules after waiting for 100 seconds. Half of electric two-wheelers cannot endure 49.0 seconds or longer at red-light phase. Red phase time, motor vehicle volume, and conformity behavior have important effects on riders' waiting times. Waiting endurance times would decrease with the longer red-phase time, the lower traffic volume, or the bigger number of other riders who run against the red light. The proposed model may be applicable in the design, management and control of signalized intersections in other developing cities.

## 1. Introduction

With the social and economic development and the rapid increase of motor vehicles, traffic problems become increasingly serious in many cities, and more and more attentions are paid to traffic research [1, 2]. Nonmotorized vehicles (i.e., mainly regular bicycles and electric two-wheelers) are one of the most popular modes of transportation in some Asian developing countries, such as India, Vietnam, Cambodia, and China. Even in developed countries, cycling travel is recognized as low energy consumption, healthy to the users, and does not damage the health of others. The Canadian Census indicates a 42% increase in daily bike commutes between 1996 and 2006. The U.S. Census Bureau reports almost twice as many bike commuters in 2009 as in 2000 [3].

**Figure 1:**
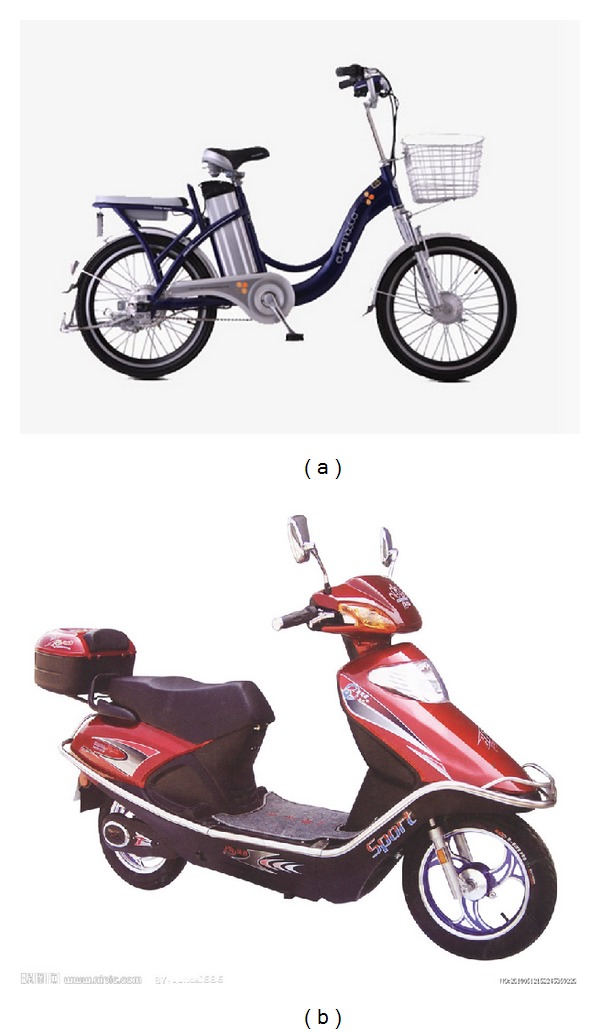
Bicycle-style in the left and scooter-style in the right.

In recent ten years, electric bike (e-bike) has entered people's life. Due to its labor-saving and speed, e-bike has emerged as a popular mode of transportation in many large cities in China. Electric two-wheeler use has rapidly expanded in China, in the process changing the mode split of many cities [4]. In 2012, the number of Chinese e-bikes was about 140 million [5]. Electric two-wheelers in China have relatively low speeds and weights compared to a motorcycle. Both bicycle-style electric two-wheelers (with functioning pedals) and scooter-style electric two-wheelers (with many of the features of gasoline scooters) are classified as bicycles and are given access to bicycle infrastructure (see Figure 1).

However, the growing popularity of cycling traffic also entails safety concerns as observed in accident and injury statistics. With the rapidly increasing number of electric two-wheelers, more and more people pay much concern about traffic security problems involved with electric two-wheelers. In 2004, 589 electric two-wheelers died and 5295 seriously injured in road accidents [6]. In 2010, the corresponding figures increased to 4029 dead and 20, 311 seriously injured, respectively, representing 6.2% of all traffic fatalities and 8.0% of injuries [7].

Previous research on pedestrians also points to several variables of interest regarding violation behaviors. For example, Keegan and O'Mahony gave reports about pedestrians' street-crossing behavior influenced by travel distance and waiting time [8]. Other researchers paid much attention to the influences of personal features on the street-crossing behavior [9–11]. Some useful reviews of the existing research on pedestrian street-crossing behavior in urban roads can be found in Ishaque and Noland [12] and Papadimitriou et al. [13].

Unfortunately, only a few studies have investigated the violation behavior of riders, much less to electric two-wheelers. Johnson et al. identified three distinct types of violated cyclists that are exposed to different levels of risk: racers, impatients, and runners [14]. Johnson et al. used questionnaire survey to investigate the reasons for Australian cyclists' red-light infringement [15]. Wu and Zhang used logistic model to study cyclist red-running behavior in China [16, 17]. In addition, some researchers investigated travel characteristics, mode shift, riding practices, risk-taking behavior, environmental impacts, and user safety perceptions of electric two-wheelers [18–22]. Till now, however, no literature is involved with waiting endurance times of electric two-wheelers.

Riders must apply greater caution when crossing streets and waiting to cross because they are more likely to be injured in the car-rider conflict [23]. Especially, the process of waiting to cross is crucial for riders' crossing. Once riders terminate the waiting processed in the red-light phase, they would violate the traffic rules and put themselves in danger. Riders' waiting processes can be considered as time-continued states which are influenced by personal characteristics and traffic conditions.

In this paper, a hazard-based duration approach is adopted to describe waiting endurance times of electric two-wheelers at signalized intersections. Duration models have been used extensively in biometrics and reliability engineering for decades [24]. Duration models can be used to determine causality in duration data and they are also useful tools in the field of transportation [25–29]. Hazard-based duration models of survival analysis have an advantage in that it allows the explicit study of the relationship between duration time and the explanatory variables. More importantly, duration models can deal with not only uncensored data but also censored data. For example, the exact waiting duration reflecting rider endurance cannot be observed if riders could wait until the permission of traffic rules. Accordingly, the waiting times of electric two-wheelers are modeled by a Cox proportional hazard model. The covariates related to personal features and traffic conditions are investigated to capture the influenced factors of rider behavior. The results give the waiting time when a rider is likely to cross the red light and the significantly influential factors on waiting endurance time.

## 2. Method

### 2.1. Duration Model

The variable of interest in duration model is the survival time that elapsed from the beginning of an event until its end. The waiting time of an electric two-wheeler at red light can be regarded as the waiting duration that starts when a rider arrives at the intersection at the red period and ends when the rider begins to cross the intersection.

Let *T* denote the survival time. In waiting time analysis of electric two-wheelers, the survival time is the waiting duration of a rider at a signalized intersection. It is assumed that the waiting duration starts when a rider of electric two-wheeler arrives at the intersection at the red period and ends when the rider begins to cross the intersection.

Then, the survival function is denoted by *S*(*t*). It is also called endurance probability or survivor probability in duration literature. It represents the probability that the duration does not elapse before time *t* as follows:

(1) $$\begin{array}{c} S\left(t\right)=\text{Pr}\left(T>t\right). \end{array}$$

In this paper, it is defined as the probability of the adherence to traffic rules with waiting time *t*, or the probability that a rider can endure waiting for a time period *t*.

The failure probability, or violating probability of riders' crossing behavior, is then

(2) $$\begin{array}{c} F\left(t\right)=\text{Pr}\left(T\leq t\right)=1-S\left(t\right) \end{array}$$

which is known as the cumulative distribution of *T*.

The waiting duration time *T* has a probability density function which is defined as the limit of the probability of failure in a small interval per unit time. It can be expressed as

(3) $$\begin{array}{c} f\left(t\right)=\frac{\partial F\left(t\right)}{\partial t}=\underset{\Delta t\to 0}{\lim}\frac{P\left(t\leq T<t+\Delta t\right)}{\Delta t}. \end{array}$$

The density function is also known as the unconditional failure rate.

The hazard function *h*(*t*) of duration time *T* gives the conditional failure rate. In this study, the hazard function is the instantaneous rate at which the waiting duration will end in an infinitesimally small time period, Δ*t*, after time *t*, given that the duration time has lasted to time *t* seconds:

(4) $$\begin{array}{c} h\left(t\right)=\underset{\Delta t\to 0}{\lim}\frac{\text{Pr}\left(t\leq T<t+\Delta t\;|\;T\geq t\right)}{\Delta t}=\frac{-d\ln S\left(t\right)}{dt}. \end{array}$$

To accommodate the effects of exogenous variables or systematic factors is an important characteristic of duration models. These variables or factors are called covariates. A covariate is an independent variable not manipulated by experiment or environment but still can influence the duration. Using this hazard function, the effects of covariates on the waiting duration time can be introduced. To include covariates that affect duration time, the Cox proportional hazard model is widely used [30]. This is defined as

(5) $$\begin{array}{c} h\left(t\right)=h_{0}\left(t\right)\exp\left(\beta^{\prime }Χ\right), \end{array}$$

where *h*_0_(*t*) is called the baseline hazard function and can be interpreted as the hazard function when all covariates are ignored. exp⁡(**β**′**Χ**) is a commonly used functional form for covariate effects, where **Χ** = (*x*_1_,*x*_2_,…,*x*_*p*_)′ is a set of covariates and **β** = (*β*_1_, *β*_2_,…, *β*_*p*_) is a vector of estimable coefficients. These coefficients can be estimated from the observed data and indicate the magnitude of the effects of their corresponding covariates.

Combining (4) and (5), the survival function can be written as

(6) S(t)=exp⁡[−∫0th(w)dw]={exp⁡[−H0(t)]}exp⁡⁡(β′Χ),

where *H*_0_(*t*) = ∫_0_^*t*^ *h*_0_(*w*)*dw* represents the baseline cumulative hazard function. Thus, the covariates can be incorporated into the survival function.

### 2.2. Model Estimation

The main interest of this paper is to identify from the *p* covariates a subset of variables that affect the violation hazard more significantly and, consequently, the waiting duration time at a signalized intersection. We are concerned with the regression coefficients. If *β*_*i*_ is zero, the corresponding covariate is not related to the waiting time. If *β*_*i*_ is not zero, it represents the magnitude of the effect of *x*_*i*_ on hazard when the other covariates are considered simultaneously.

To estimate the coefficients, *β*_1_, *β*_2_,…, *β*_*p*_, a partial likelihood method is adopted. Suppose that *k* of the waiting duration times from *n* electric two-wheelers are uncensored and distinct, and *n*-*k* are right-censored. Let *t*_(1)_ < *t*_(2)_ < ⋯<*t*_(*k*)_ be the ordered *k* distinct duration times with corresponding covariates **x**_(1)_, **x**_(2)_,…, **x**_(*k*)_. Let **R**(*t*_(*i*)_) be the risk set at time *t*_(*i*)_. **R**(*t*_(*i*)_) consists of all riders whose duration times are at least *t*_(*i*)_. For the particular duration time *t*_(*i*)_, conditionally on the risk set **R**(*t*_(*i*)_), the probability is

(7) $$\begin{array}{c} \frac{\exp\left(\sum_{j=1}^{p}\beta_{j}x_{j(i)}\right)}{\sum_{l\in R(t_{\left(i\right)})}\exp\left(\sum_{j=1}^{p}\beta_{j}x_{jl}\right)}=\frac{\exp\left(\beta x_{\left(i\right)}\right)}{\sum_{l\in R(t_{\left(i\right)})}\exp\left(\beta x_{l}\right)}. \end{array}$$

Each distinct duration time contributes a factor and hence the partial likelihood function is

(8) $$\begin{array}{c} L\left(\beta \right)=\overset{k}{\underset{i=1}{\prod }}\frac{\exp\left(\beta x_{\left(i\right)}\right)}{\sum_{l\in R(t_{\left(i\right)})}\exp\left(\beta x_{l}\right)} \end{array}$$

and the log-partial likelihood is

(9) $$\begin{array}{c} l\left(\beta \right)=\log L\left(\beta \right)=\overset{k}{\underset{i=1}{\sum }}\left\{\beta x_{\left(i\right)}-\log\left[\underset{l\in R(t_{\left(i\right)})}{\sum }\exp\left(\beta x_{l}\right)\right]\right\}. \end{array}$$

The overall goodness-of-fit of the model estimation is determined by the likelihood ratio (LR) statistics, which is specified as

(10) $$\begin{array}{c} X_{L}=2\left[l\left(\hat{\beta }\right)-l\left(\beta_{0}\right)\right], \end{array}$$

where *l*(**β**_0_) is the log-partial likelihood for null model with all the regression coefficients set as zero and $l(\hat{\beta })$ is the log-partial likelihood at convergence with *p* regression coefficients. The estimation of *H*_0_(*t*) and other detailed statistical presentations of duration models can refer to Lee and Wang [24].

## 3. Data

### 3.1. Site Survey Design

A cross-sectional observational study was conducted at six signalized intersections in Beijing. Three criteria were used to select the observational sites. First, the selected sites should represent the typical intersection design characteristics and traffic conditions of urban areas in Beijing. Second, the selected intersections should have similar characteristics involved with geometric, traffic conditions, traffic control, and the absence of pointsmen. In addition, there have to be a reasonably high number of electric two-wheelers traffic during the observation period.

Video cameras were used to collect data of bicyclists' crossing behaviors at signalized intersections. The cameras were carefully placed so that the road users were unaware that they were being observed. The data collection was conducted on weekdays during daylight hours (i.e., 8:00 a.m. to 5:30 p.m.) in good weather conditions.

To record the waiting durations of electric two-wheelers, all road users who entered the intersection were recorded on video, but only the riders arriving in red-light phases were coded. Only the electric two-wheelers who arrived in the red-light period were defined as valid sample. In addition, left turners and right turners were excluded because of the limited field of view of the cameras. The waiting duration was from the time a rider of electric two-wheeler arrived at the crossing location to the time he/she began to cross. It can be classified into two kinds: uncensored data and censored data. The uncensored data was defined as the waiting duration which ended within the red-light period (violating crossing). Otherwise, the waiting duration was called the censored data as long as it ended within the green-light period (normal crossing). For censored data, the exact waiting duration which can reflect waiting endurance times of electric two-wheelers is unknown.

### 3.2. Covariate Selection

The covariate selection takes into account the previous researches and arguments regarding the effects of the exogenous variables and human factors on rider violation behavior. Two broad sets of variables are considered as covariates: personal characteristics and traffic conditions. Personal characteristics include age and gender. The selected covariates of traffic conditions include riders' waiting positions, violating number upon arrival, red-phase time, and crossing traffic volume. The practical effects on waiting behavior and the feasibility of data acquisition are considered in the covariate selection. The following covariates, as shown in Table 1, are adopted to construct the duration model.

### 3.3. Descriptive Statistics

Of the 961 valid observations, 560 (58.27%) electric two-wheelers violated the traffic regulations. The average waiting time of all samples was 34.1 seconds, with a standard deviation of 33.5 seconds. The average waiting time of the violating crossing was 25.6 seconds while the average waiting time of the normal crossing is 46.7 seconds. The maximum waiting duration was 174 seconds while the minimum was 0 second. The latter means that electric two-wheelers cross the intersection without any wait. This descriptive statistic cannot reflect the exact waiting behavior due to the neglect of the censored data. The estimation of the waiting endurance times with censored data will be discussed later.

## 4. Empirical Results

The results are discussed in two subsections. The overall results are presented in the first subsection including model fit statistics and survival probability estimation. The second subsection presents the effects of covariates.

### 4.1. Overall Results

**Figure 2:**
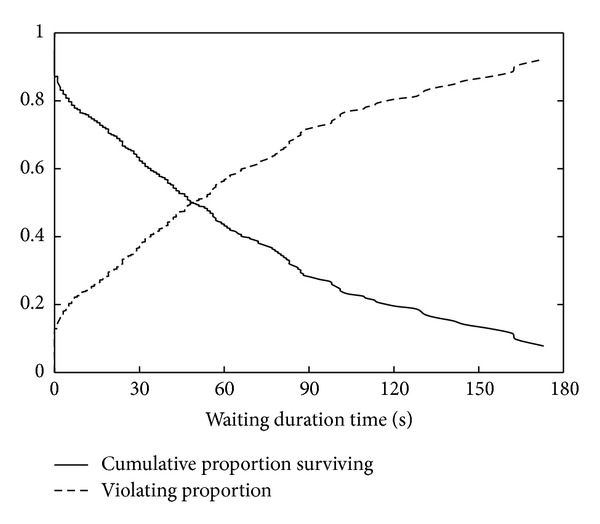
Cumulative proportion surviving versus waiting duration time.

(1) Model Fit Statistics. The LR statistic of the estimated model clearly indicates the overall goodness-of-fit (the LR statistic is 179.8, which is greater than the chi-squared statistic with 8 degrees of freedom at any reasonable level of significance). The significant level corresponding to each covariate is given by *P* value in Table 2. From the results, most of the included covariates are statistically significant at the 0.10 level of significance. It means these covariates are significantly related to waiting endurance times. Only age and gender are identified as insignificant predictor variables on waiting duration times. The significance level of each covariate suggests that the importance of covariate should be interpreted carefully. Furthermore, the forward selection method was used to analyze the covariates' degree of importance. The result shows that important ranking of significant factors was waiting position, violating number, traffic volume, and red-phase time, respectively. Finally, the regression equation with significant variables (*P* < 0.05) obtained is as follows:

(11) log⁡h(t)h0(t)=0.177xWP1+1.004xWP2+0.076xCN−0.159xMV+0.004xRT.

(2) Cumulative Surviving Proportion.  Figure 2 (solid line) gives the cumulative surviving proportion calculated by the duration model, which represents the proportion complying with the traffic rules while waiting at signalized intersections. Correspondingly, the dashed line represents the violating proportion of electric two-wheelers. The violating proportion for estimated model presents a general rising trend with elapsed waiting duration (dashed line in Figure 2). The violating proportion can be divided into three parts according to the gradient. Firstly, a sharp rise for the short duration indicates that there are a number of electric two-wheelers who would violate to cross without any delay. About 12.8 percent of riders can be defined as risk preferences since they show high violation inclination and very low waiting endurance (<1 seconds). Then, the curve rises smoothly from 1 second to 100 seconds. This steady increase reflects the number of rider violations is increasing continuously. The rising trend of violating proportion indicates that the red-light running behavior of most riders is time dependent. It means that riders are easy to end waiting duration and violate the traffic rules with the elapsed duration. When the waiting duration time is larger than 100 seconds, the curve rises slightly. It means that there are 25.0% percent of riders who can endure 100 seconds, or longer, and they are generally nonrisk takers. Finally, about half of electric two-wheelers observed cannot endure 49.0 seconds or longer.

### 4.2. Analysis of Covariate Effects

In the Cox proportional hazard model, the effects of covariates are multiplicative on the baseline hazard function. A negative coefficient on a covariate implies that an increase in the corresponding covariate decreases the hazard rate or, equivalently, increases the waiting duration. Furthermore, when a covariate changes by one unit, the hazard would change by [exp⁡(*β*) − 1] × 100%.

The effect of gender (GEN) shows that male riders have shorter waiting endurance time and higher tendency to cross against a red light. Male riders were 1.159 times more likely to have red-light infringement behavior than female riders. Zhang and Wu reported that male riders are 1.265 times more likely than females to have shorter waiting times [17], and other qualitatively similar results in pedestrian study were obtained by Hamed [31] and Tiwari et al. [32].

The effect of age (AGE) indicates that older electric two-wheelers have longer waiting time. Young riders were 1.248 times more likely to run against a red light upon approaching the intersection than elderly riders. Middle-aged riders were 1.191 times more likely to run against a red light compared to elderly riders. This is partly because elderly riders have stronger risk consciousness of traffic violations. In addition, elderly riders' trip purposes are seldom related to work or school so they are not in a hurry.

The effect of waiting position (WP) shows that electric two-wheelers approaching the motorized lane have shorter waiting time and high red-light running risk. Electric two-wheelers at the position close to the motorized lane were 2.729 times more likely to run against a red light than those who waited in the appropriate position. Riders in the middle position were 1.193 times more likely to violate traffic rules compared to riders in the appropriate position. This is because riders' waiting positions, to some extent, reflect their risk preference. Riders who are near to the motorized lane are likely to have high risk tendency. They may be more anxious to cross the intersection than those who wait in the appropriate position. Most of riders approaching the motorized lane were risk takers, and those riders in the appropriate position were generally nonrisk takers.

The effect of covariate VN (violating number) reflects the conformity behavior of electric two-wheelers when approaching the intersection. Conformity psychology reveals that people may follow other's crossing action including traffic violation. The electric two-wheelers who are apt to be affected by other riders have shorter waiting duration time compared to those with low conformity psychology. Moreover, a group of riders who crosses together could increase the risk of violation, because the street-crossing behavior shows obvious groupment and conformity [10]. With the increasing number of riders in a group and longer waiting time, riders are easy to be influenced by each other, especially the red-light running behavior.

The covariate of RT (red-phase time) has a positive impact on violation risk. Waiting times of electric two-wheelers increases with the longer red-phase time. Riders may become more impatient and they are apt to end waiting duration. In the site survey, many electric two-wheelers began to cross at the left-turn phase for vehicles, and they ignored the fact that the rider signal was still red. In our duration model, the lower bound of the confidence interval for red phase time is only slightly above 1. This suggests that the importance of red-phase time should be interpreted carefully.

The covariate of MV (motor vehicle volume) has a distinct negative effect on the risk of red-light running behavior. Electric two-wheelers are likely to wait longer times under high traffic flow conditions compared to those under relative low traffic flow conditions when other factors are controlled. The average headways between successive motorized vehicles under low traffic flow conditions are larger than those under high traffic flow conditions. Therefore, riders under low traffic flow conditions are likely to have more chance to cross the intersection than those under high flow conditions.

## 5. Model Application

The model formulated in this paper can be applied to forecast temporal shifts in waiting duration times of electric two-wheelers due to changes in traffic flow, traffic management, and control. This is particularly relevant today because of changes of traffic modes and traffic volumes. For instance, with the social and economic development, private cars increase rapidly in recent decades in China. At the same time, electric two-wheelers are more and more prevalent in some Chinese cities with the technology progress. Similarly, red-phase times and traffic volumes at signalized intersections may be changed with the construction of a new business district or the temporal traffic control during major events and incidents. All of these changes will have an effect on waiting duration times of electric two-wheelers, and the waiting duration model in this paper can be used to assess these impacts and provide reliable information regarding the temporal distribution of waiting duration times at signalized intersections. In the next paragraphs, two most important variables in predicting waiting duration times at red-light phase are taken as examples to present the model application.

**Figure 3:**
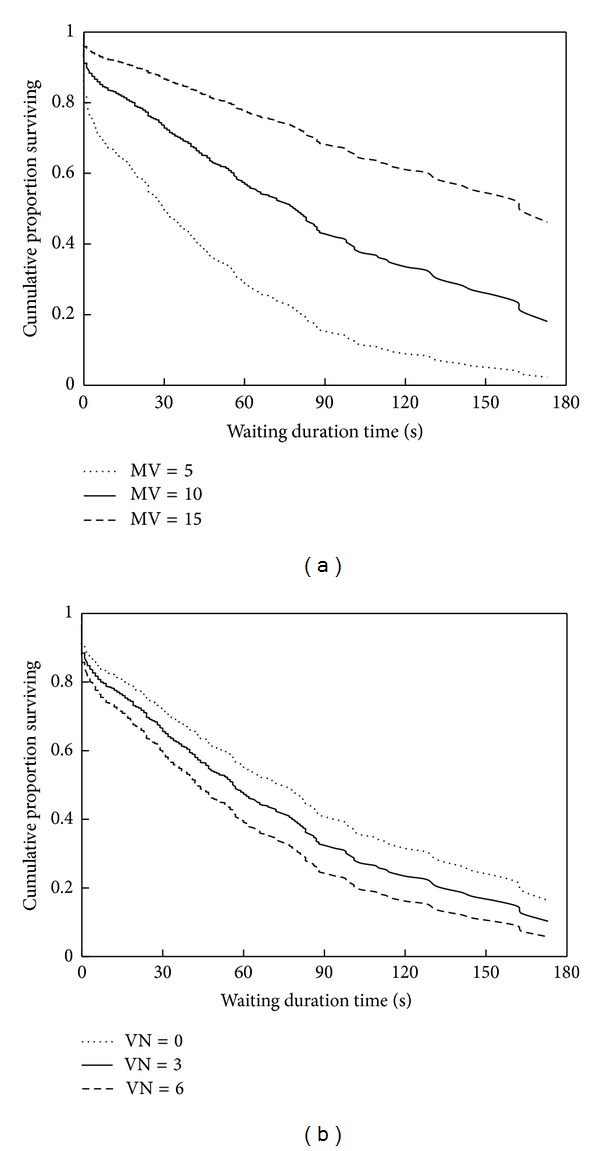
Waiting endurance time distributions with different (a) traffic volumes and (b) violating riders.

Firstly, Figure 3(a) gives the new distributions of waiting duration times due to the change in motor vehicle volume. The differences between the curves indicate the significant effect of motor vehicle volume on waiting endurance time when other variables are controlled and their values are the average conditions of all samples. While traffic flow increases from 5 to 10 and 15 veh·min^−1^, the medians of waiting duration time are 30.5 s, 79.1 s, and 162.6 s; the 75% quartiles are 3.5 s, 28.2 s, and 72.0 s. It means that only 25% of electric two-wheelers can endure 3.5 seconds or longer when motor vehicle volume is 5 veh·min^−1^. Meanwhile, 25% of electric two-wheelers can endure 72.0 seconds or longer when motor vehicle volume is 15 veh·min^−1^. Here, the 50% quantile is defined as the approximate waiting endurance time. Waiting endurance times of electric two-wheelers would be 2.06 times with 15 veh·min^−1^ compared to those with 10 veh·min^−1^ (162.6 versus 79.1).

Analogously, other variables estimated by the duration model can be used to predict the distribution of waiting duration time.  Figure 3(b) gives the effect of violating riders after arrival on waiting endurance times of electric two-wheelers. While the number of violating riders increases from 0 to 3 and 6, the medians of waiting duration time are 74.0 s, 56.7 s, and 43.5 s. 50% of electric two-wheelers can endure 74.0 s or longer when there are no violating riders after arrival. The result indicates that waiting endurance time is 1.70 times with no violating riders compared to those with 6 violating riders (74.0 versus 43.5).

The hazard-based duration methodology can capture the effects of covariates on waiting duration times of electric two-wheelers at intersections. Before the applications, however, it is noted that the model should be estimated using the specified field data. Additionally, the explanatory variables should be chosen flexibly according to the research objective and the traffic reality.

## 6. Conclusions

This paper investigated the waiting endurance times of electric two-wheelers at signalized intersections through the data acquired in Beijing, China. Waiting endurance times of electric two-wheelers were estimated by using a Cox proportional hazard model.

The paper provides several important insights into the determinants of red-light running behavior of electric two-wheelers, especially the relation between waiting endurance time and violation proportion. First, the results indicate that the violation risk of electric two-wheelers is time dependent. As signal waiting time increases, electric two-wheelers get impatient and violate the traffic signal. With the longer waiting duration times of electric two-wheelers, they are more likely to end the wait soon. Second, some crucial time points deserve our concern: 1 second and 100 seconds. The 1 second indicates electric two-wheelers who are at high risk of red-light running without any delay. They account for 12.8% of the sample in the study. The duration of 100 seconds reflects the riders' endurance. About 25.0% percent of electric two-wheelers can obey the traffic rules after waiting for 100 seconds. These riders are generally nonrisk takers. Third, red-phase time, motor vehicle volume, and conformity behavior have important effects on waiting endurance times of electric two-wheelers. Minimizing the effects of unfavorable condition may be an effective measure to obtain conscious cooperation and behavioral changes of electric two-wheelers. Finally, the model formulated in this paper can be applied to forecast temporal shifts in waiting duration times of electric two-wheelers due to changes in traffic flow, traffic management, and control.

In terms of the future work, the behavior difference between electric bike and common bike at signalized intersections should be investigated. Next, the respective influence proportions of the factors to waiting endurance time should be discussed. In addition, the risk preference of electric two-wheelers needs to be studied by questionnaire survey. It is hoped that these findings may give better understanding of behavioral characteristics of electric two-wheelers and be applicable in the traffic design, management, and control of signalized intersections under mixed traffic conditions.

## Figures and Tables

**Table 1:** Covariates selection and explanation.

Covariate	Type	Explanation
AG (age group)	Categorical variable	0 if less than 30 (young), 1 if 30–50 (middle-aged), 2 if more than 50 (elderly),
GEN (gender)	Categorical variable	1 if male, and 0 if female
WP (waiting position)	Categorical variable	0 if appropriate position in nonmotorized lane (appropriate), 2 if close to motorized lane (nearest), and 1 if between the two (middle)
VN (violating number)	Continuous variable	The number of other cyclists who violate against the red light after the rider arrives
MV (motor vehicle volume)	Continuous variable	Average motor vehicle volume per lane per min on red-light phase when the rider arrives
RT (red phase time)	Continuous variable	The period in the signal cycle during which the signal is red for riders

**Table 2:** Estimation in waiting duration model.

Variable	Coefficient (*β*)	Standard error	Wald value	*P* value	Exp. (*β*)	95% CI for Exp. (*β*)	
						Lower	Upper
GEN	0.148	0.118	1.575	0.209	1.159	0.920	1.460
AGE			1.250	0.535			
Young versus elderly	0.221	0.199	1.243	0.265	1.248	0.845	1.842
Middle-aged versus elderly	0.174	0.188	0.865	0.352	1.191	0.824	1.720
WP			95.820	<0.001			
Middle versus appropriate	0.177	0.152	1.345	0.246	1.193	0.885	1.609
Nearest versus appropriate	1.004	0.134	55.817	<0.001	2.729	2.097	3.552
CN	0.076	0.015	26.369	<0.001	1.079	1.048	1.111
MV	−0.159	0.036	19.995	<0.001	0.853	0.796	0.915
RT	0.004	0.002	4.859	0.027	1.004	1.000	1.007
